# Influence of Vinasse Application in the Structure and Composition of the Bacterial Community of the Soil under Sugarcane Cultivation

**DOI:** 10.1155/2016/2349514

**Published:** 2016-07-26

**Authors:** Wellington Pine Omori, André Ferreira de Camargo, Karla Cristina Stropa Goulart, Eliana Gertrudes de Macedo Lemos, Jackson Antônio Marcondes de Souza

**Affiliations:** ^1^Department of Biology Applied to Agriculture and Livestock, School of Agricultural and Veterinarian Sciences, São Paulo State University (Univ Estadual Paulista), Paulo Donato Castellane Km 05 Road, 14884-900 Jaboticabal, SP, Brazil; ^2^Department of Technology, School of Agricultural and Veterinarian Sciences, São Paulo State University (Univ Estadual Paulista), Paulo Donato Castellane Km 05 Road, 14884-900 Jaboticabal, SP, Brazil

## Abstract

Although the use of vinasse as a waste helps replenish soil nutrients and improves the quality of the sugarcane crop, it is known that vinasse residues alter the diversity of bacteria naturally present in the soil. The actual impacts of vinasse application on the selection of bacterial taxa are not understood because no studies have addressed this phenomenon directly. Analysis of 16S rRNA gene clone sequences from four soil types showed that the soil planted with sugarcane and fertilized with vinasse has a high diversity of bacteria compared to other biomes, where Acidobacteria were the second most abundant phylum. Although the composition and structure of bacterial communities differ significantly in the four environments (Libshuff's test), forest soils and soil planted with sugarcane without vinasse fertilizer were similar to each other because they share at least 28 OTUs related to Rhizobiales, which are important agents involved in nitrogen fixation. OTUs belonging to Actinomycetales were detected more often in the soil that had vinasse applied, indicating that these groups are more favored by this type of land management.

## 1. Introduction

Research on bacterial diversity in soils planted with sugarcane (*Saccharum* spp.) has indicated that the greater portion of these microorganisms is unknown [1–3]. The importance of the study of bacterial populations in soil is based on approaches depending on the cultivation because these microorganisms could provide new biological resources for the production of commercial inoculants. The bacteria are used in studies that provide insight into mechanisms of the production of compounds that increase sugarcane yield, such as siderophores, indolic components, biological nitrogen fixation, and inorganic phosphate solubilization [3]. Beyond their importance in agronomical applications, Plant Growth-Promoting Bacteria (PGPB) can mitigate the health of contaminated ecosystems when used for bioremediation of a contaminated site [4].

The reuse of agricultural organic waste promotes conservation of natural resources and helps in carbon recycling and recycling of other mineral elements [5, 6]. Based on specific recommendations, reusing these wastes can improve soil quality and reduce dependence on commercial fertilizers that are considered hazardous [7]. Despite its importance for replenishing soil nutrients, vinasse can have a serious environmental impact if used improperly [8] because an excessive accumulation of nutrients can cause contamination [4]. The heterogeneity of vinasse composition contraindicates its use as an organic fertilizer for many reasons, the most common of which is the leaching of potassium nitrate that contaminates the groundwater [7, 9]. Although the effects of fertilization with vinasse on the physicochemical properties of soil have been studied for a long time [7–9], little is known about its impact on the bacterial community.

Such research has been nonspecific because indirect techniques to assess local microbiota such as respirometry are generally used [10]. It is commonly known that the use of vinasse, compared to the application of other agricultural wastes, contributes to improvement of the biological quality of the soil because of an increase in the total bacterial population, in particular of group Actinomycetes [7].

The use of the molecular techniques based on 16S ribosomal RNA (16S rRNA) gene clone library allows an indirect assessment of the bacterial community, composing the bacterial community of soils subjected to different agricultural management practices, and of the impacts on the composition and diversity of different bacterial groups [1, 2, 11]. Considering that the major part of the bacterial population in soil cannot be cultivated by traditional techniques [5, 12] and play a crucial role in the maintenance of different ecosystems [2, 13–15], these microorganisms are considered as biological indicators of soil quality [7].

This study aimed to identify the bacterial communities in soils in which sugarcane was cultivated and subject to different management practices using the analysis of partial sequences of clones from 16S rRNA gene libraries.

## 2. Materials and Methods

### 2.1. Description of the Local Experiments and Soil Samples

All samples were collected in duplicate at 0–20 cm depth (393 mL). The points were randomly chosen from the central point of each area of interest: (1) soil on which sugarcane was cultivated that was organically fertilized with vinasse (SPV); (2) a heterogeneous soil that contained the master vinasse channel (SMC); (3) soil on which sugarcane was cultivated and burned before harvesting (SPS), and (4) a secondary forest soil (SFS).

Soil sampling of SPV, SMC, and SFS was performed in February 2010 at the Itaquerê Farm (upcountry São Paulo, Brazil) in Nova Europa city. RB965911 (4° cutting stage) was cultivated on SPV (502 m altitude, 21°49′41,07′′S and 48°36′11,46′′O). Vinasse was applied to this soil and the harvest was fully mechanized since 2008 until the time of sampling. SMC (480 m altitude, 21°49′35,17′′S and 48°36′41,06′′O) had not been used to transport vinasse for approximately 15 days because the harvest was finished. The master channel openings were made in the soil and were used to transport liquid vinasse produced in distilleries adjacent to the sugarcane area by gravity in artificial streams. The soil was irrigated with vinasse via motorized pumping from the master channel. Due to the constant shifting of vinasse to different cultivation areas when transported in SMC, soil particulates accumulate and form a mixture of different soils with organic waste from the vinasse and crop residues. SFS (475 m in altitude, 21°48′32,77′′S and 48°36′23,68′′O) was located near the sugarcane cultivation areas, and because it was at a lower altitude, SFS was subject to anthropogenic perturbations of crop management, mainly due to the accumulation of soil and nutrients carried by rainwater. SPS was sampled in January 2013 at the Pau D'alho Farm (Bebedouro City). This area is located at 580 m of altitude (20°55′59,90′′S and 48°25′49,36′′) and was planted in sugarcane with manual handling after harvesting the burned sugarcane.

According to the Koeppen climatic classification, Nova Europa and Bebedouro which are located northwest of São Paulo state have warm weather, a tropical rainy climate, a dry winter, and colder months (Aw). Nova Europa city has an annual median temperature of 29.3°C and precipitation of 1,341.4 mm and Bebedouro has a temperature of 31.0°C and precipitation of 1,333.8 mm.

After sampling, a portion of each of the four soils was forwarded to the Laboratory of Analysis of Soils (Faculdade de Ciências Agrárias e Veterinárias de Jaboticabal/SP, UNESP, Brasil) to obtain the physical-chemical parameters. The soil properties are shown in Table 1.

### 2.2. DNA Extraction, PCR, and Cloning

The total DNA from each soil sample was obtained using a SPIN Kit for Soil® (MP Biomedicals), according to the manufacturer's instructions. 16S rRNA gene was partially amplified (variable regions 1 and 2) with Y1 and Y2 primers [16], yielding Polymerase Chain Reaction (PCR) products of approximately 300 bp. The PCR reaction mixture consisted of 20–50 ng/*μ*L of DNA, 5.0 pmol/*μ*L of each primer, 0.2 mM of dNTPs, 3.0 mM of MgCl_2_, Buffer 1x, and 2.5 U* Taq* DNA polymerase (Ludwig Biotec). A thermocycler model PTC-100*™* Programmable Thermal Controller (MJ Research, Inc.) used a thermal profile of 95°C for 2 min, 35 cycles of 95°C for 45 s, 65°C for 45 s, and 72°C for 1 min, and 30 s ending with 72°C for 5 min. After the PCR reaction, the products were purified with a Wizard® SV Gel and PCR Clean-Up System (Promega) and for PCR cloning CloneJET*™* Cloning Kit (Fermentas) was used as per the manufacturer's instructions.

Colonies in Petri dishes were collected with a sterile wooden toothpick and organized into 96-well ELISA plates containing 100 *μ*L of Luria-Bertani medium with ampicillin (50 *μ*g/mL). After overnight development of the clones at 37°C, a copy of each library was inoculated to extract the plasmid DNA [17] and 100 *μ*L of 40% glycerol (v/v) was added to the original library for storage at −80°C.

### 2.3. Sequencing and Taxonomic Evaluation

Amplicons of 16S rRNA gene were sequenced using Y1 primer. DNA sequences were obtained with ABI PRISM Big Dye Terminator cycle sequencing-ready reaction kit (Applied Biosystems) using ABI 3100 (Perkin Elmer) capillary sequencer, following the manufacturer's instructions. The sequences used for the analyses were those with quality Phrep ≥ 20 [18]. A search for chimeric sequences was performed with Decipher software [19] and taxonomic identification to the level of phylum, class, order, family, and genus was made with the Classifier software using confidence limits of 80, 85, 91, 92, and 95% [11], respectively. Sequences that scored 95% confidence in the Classifier were used to search for similar sequences in MegaBlast (16S rRNA gene of the Bacterial and Archaea) [20] from the National Center for Biotechnology Information (NCBI) or the SeqMatch Ribosomal Database Project (RDP II) [21]. These searches were conducted in March 2016.

### 2.4. Construction of the Classification Phylogenetic Tree

The sequences of 16S rRNA gene were submitted to GenBank under the following accession numbers: SPV (KJ749164–KJ749443), SMC (KJ749444–KJ749650), SPS (KJ748893–KJ749163), and SFS (KJ748700–KJ748892). The sequences from the four ecosystems (SPV, SMC, SPS, and SFS) (MegaBlast or SeqMatch) were used to construct the phylogenetic tree. The database sequences that were most similar to the sequences of clones from this study were used to compare the phylogenetic tree after recovery from the GenBank/National Center for Biotechnology Information (NCBI). Alignment was performed based on ClustalW method [22] available on BioEdit 7.2.3 [23]. The distance matrices were calculated from the sequence alignment program using Dnadist from the package program PHYLIP v3.6 [24] using the matrix of nucleotide substitutions of Jukes and Cantor [25] for the correction of mutations. MEGA 6.06 [26], a neighbor-joining method [27], bootstrapping with 1,000 replications and an optional pairwise deletion, was used for the construction of the phylogenetic tree.

### 2.5. Diversity Estimation, Comparison of the Library, and Statistical Analysis

*α*-diversity was obtained by analyzing the sequences of all clones (SPV, SMC, SPS, and SFS) followed by the construction of a rarefaction curve and obtaining the indices of richness, evenness, Shannon (*H*), and Chao 1, considering an evolutionary distance of 3% difference (or 97% similarity). *H* was also used to estimate *γ*-diversity among the samples. Significant differences among the structure of the bacterial communities were indicated by the Libshuff test (*β*-diversity) using the Cramer-von Mises criterion (*P* ≤ 0.05). *P* value obtained was corrected with the Bonferroni correction. All data were obtained with mothur v.1.33.3 [28]. UniFrac [29] analysis was used to compare the clones of each library using a normalization step with 100 permutations, the Jackknife Environment Clusters option, and the considerate selection algorithm, which considered the relative abundance of the OTUs.

## 3. Results

### 3.1. Phylogenetic Groups Identified in Various Environments

A total of 951 partial sequences of 16S rRNA genes (free of chimeric sequences) were obtained, of which 280 were recovered from the soil on which sugarcane was cultivated and fertilized with vinasse (SPV), 271 sequences from soil on which sugarcane was cultivated (SPS), 207 from the master vinasse channel (SMC), and 193 sequences from the secondary forest (SFS). All sequences of 16S rRNA gene amplicons were combined and used for subsequent analyses. The taxonomic classification in RDP II indicated 11 different phylogenetic groups: SPV had 11 phyla, SPS had seven phyla, SMC had five phyla, and SFS had four phyla (Table 2). Proteobacteria were the most abundant phylum in the four soil types and were more frequent in SPS (63%) and SFS (60%), where Alphaproteobacteria occurred at more than 50% frequency. A few relative sequences (less than 5%) of Betaproteobacteria were identified. Gammaproteobacteria were detected only in SPS and SMC, and Deltaproteobacteria were only in SPV and SFS.

Actinobacteria and Acidobacteria were the second and third most abundant phyla in SPS and SFS, as reported in other studies [30]. Different than observed in other soils, the second most abundant phylum in SPV was Acidobacteria (28%), followed by Actinobacteria at 6% of the taxonomic affiliations. Acidobacteria and Actinobacteria showed same abundance (13-14%) in SMC; and Firmicutes (7%) phylum had the third highest frequency. Although not shared by all environments, less frequent phyla were also identified in the four environments, such as Planctomycetes, Nitrospirae, Bacteroidetes, Chloroflexi, Armatimonadetes, and TM7 (“Saccharibacteria”) (Table 2). Some clones (17% total) showed no similarity more than 80% with available sequences in RDP II; for this reason, this group was assigned to the unknown bacteria. The SPS ecosystem had fewer affiliated sequences as unclassified bacteria (5%), while the SMC was the environment showing the high number of OTUs for that group (25%) (Table 2).

**Figure 1:**
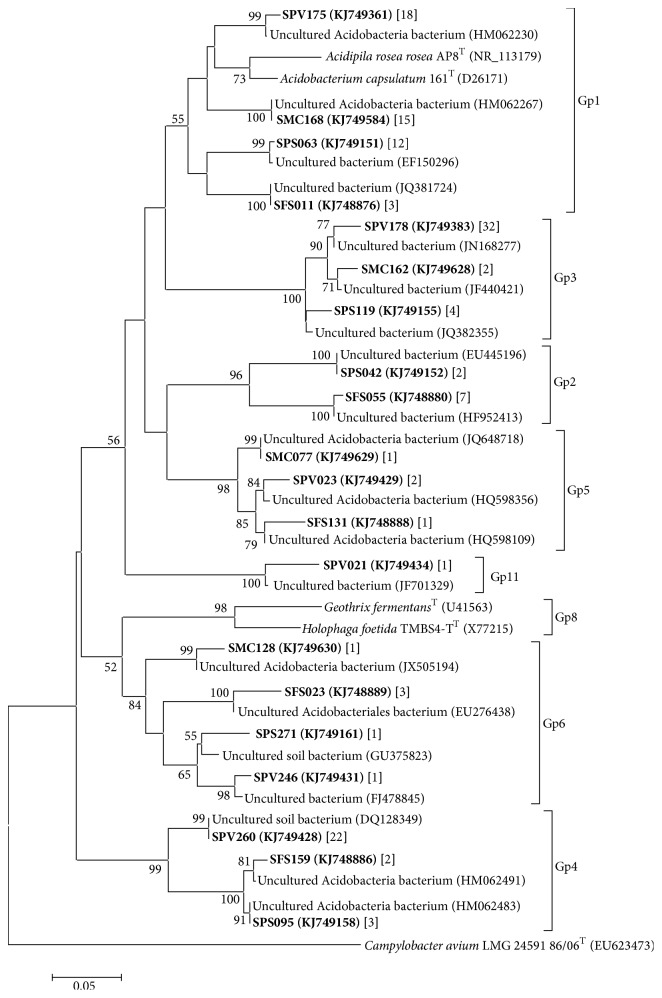
Phylogenetic tree showing species of the Acidobacteria group identified four biomes. The access numbers to the GenBank sequences obtained in this study are shown in parentheses. The numbers in brackets represent the number of sequences that were affiliated to the same taxonomic group Acidobacteria. For the tree construction was used the matrix of Jukes and Cantor nucleotide substitutions, neighbor-joining method with bootstrap for 1,000 replicas and pairwise deletion option. The nodes showed just bootstrap value of above 50%. The scale indicates 0.05 occurring nucleotide substitutions at each position. The abbreviations of the four biomes are shown in Table 1.

**Figure 2:**
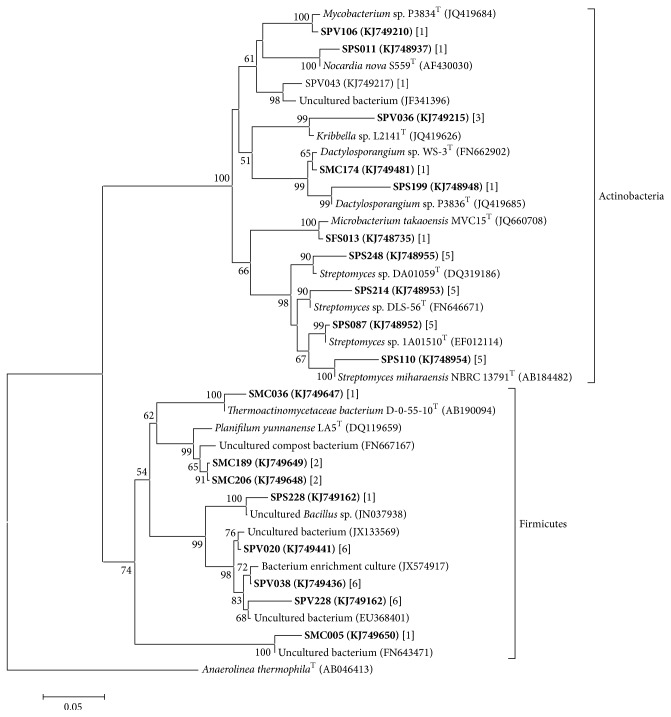
Classification phylogenetic tree showing the affiliations sequences of 16S rRNA gene for Actinobacteria and Firmicutes identified in the four biomes. The sequences obtained are shown in bold between parentheses. The numbers in brackets represent the number of sequences that were affiliated to the same taxonomic groups Actinobacteria and Firmicutes. The scale indicates 0.05 occurring nucleotide substitutions at each position. The abbreviations of the four biomes are shown in Table 1.

A high frequency of OTUs related to order Rhizobiales (Alphaproteobacteria) was found, which is a group of bacteria commonly found in soil that play an important role in biological nitrogen fixation [11]. Order Rhizobiales had different frequencies across the four biomes, identified in 109 OTUs at SPS, 73 OTUs at SFS, 39 OTUs at SPV, and 19 OTUs at SMC. All environments showed a higher taxon (Classifier), although most similar sequences were uncultured bacteria obtained from the analysis of 16S rRNA genes in the soil recovered (Figures 1 and 2).

For all soils, the formation of clusters related to subdivisions 1–6, 11, 13, and 16 Acidobacteria species in RDP II (Classifier and SeqMatch) was observed. Similar to order Rhizobiales, Gp3 and Gp4 subgroups of Acidobacteria appeared to be more sensitive to the relative frequency; as SPV represented 22–32 OTUs affiliated to species of this group, SPS, SFS, and SMC had 2–4 species (Figure 1).

### 3.2. Diversity of Bacterial Communities in Environments

The diversity environmental indices were obtained using OTUs with an evolutionary distance ≤ 0.03. All of the diversity and richness indices (Table 4), supported by the slope of the rarefaction curve (data not shown), showed the following tendency in terms of species: SPV > SPS > SFS > SMC. Although the rarefaction curve for SFS and SPS seemed to indicate a similar profile, SPS was considered to be an environment with intermediate diversity according to values obtained by Shannon and Simpson and richness estimator Chao 1 (less abundant acceptable OTUs). The less diverse environment was the SMC biome according to the diversity indices (Table 4). Because the rarefaction curve did not plateau and the slope was high for all environments (data not shown), it is evident that many new taxa could still be identified in any of these ecosystems, corroborated by the number of OTUs observed (*S*) and the values obtained by estimator Chao 1 (Table 4).

### 3.3. Effects of the Abundance of OTUs in the Structure of Bacterial Communities

**Figure 3:**
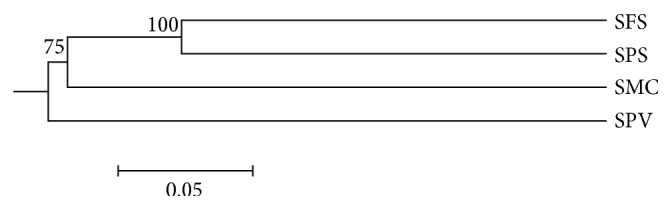
Phylogenetic tree Unifrac Jackknife Environmental Cluster analysis of 16S rRNA genes from libraries of the recovered clones from four soil biomes analyzed in this study. Node numbers indicate how often the biomes were listed as supporting the Jackknife analysis. The abbreviations of the biomes are shown in Table 1.

The Unifrac Jackknife Environmental Cluster analysis clearly shows that SPS and SFS share few taxa in common comparing the four environments (Figure 3). For 10 shared OTUs identified by mothur (97% similarity) in those environments, the relationship was almost exclusive to Alphaproteobacteria, especially those of order Rhizobiales (Tables 3 and 5). In the latter test, SMC and SPV appear to contain bacterial communities distinct from those found in SFS and SPS. Although SFS and SPS seemed to be relatively similar (Figure 3), the Libshuff molecular variance test (date not shown) indicated significant differences (*P* < 0.01). In this test, the four bacterial communities recovered from soils in directions *x*-*y* and *y*-*x* demonstrated that the sequences from each library are quite different in each type of environment.

## 4. Discussion

### 4.1. Distinction of Phylogenetic Groups Identified in Soils

Proteobacteria, a group with great morphological, physiological, and metabolic variation [31], have been found as the most abundant group in soil [30] and play an important role in the global carbon, nitrogen, iron, and sulfur cycles [5, 6, 32]. This bacterial group has been frequently reported in soils planted with sugarcane using cultivation-dependent techniques [3] and also by cultivation-independent molecular techniques [1, 2]. Although many proteobacterial strains have been isolated and characterized, an analysis based on 16S rRNA gene sequences from environmental clones and by pyrosequencing indicated that many members are still unknown, and, therefore, their specific roles in various ecosystems (marine sediments, soil, anaerobic degradation of organic components, etc.) are also unknown [5].

In our research, we found that the frequency of Proteobacteria and Actinobacteria phyla and their classes was variable depending on the type of soil management. SPV was the environment that showed the lowest frequency of those Proteobacteria in relation to SPS and SFS (Table 2). Those data demonstrate that Proteobacteria presented impaired abundance in soil under vinasse application (SPV) compared with that under burning management (SPS), in spite of the application of vinasse as a source of essential nutrients to sugarcane crop. With the exception of SMC, all of the classes of Proteobacteria had sequences that conformed to those reported in most studies on bacterial diversity in soil [11]. These observations are important because we need to document as much information as possible about the effects of vinasse on soil microbiota, once those evidences support the development of new researches and the improvement of more sustainable agricultural techniques than those available. Thus, one can promote the increase of crop productivity and improve the use of byproducts generated by sugarcane industry.

We also observed, in the four environments, sequences associated with clones assigned to Alpha-, Beta-, Gamma-, and Deltaproteobacteria (Table 2), which emphasizes that there is great potential for the exploitation of genetic resources and unknown organisms that can be accessed with the use of molecular techniques. In particular, Alpha-, Beta-, and Gammaproteobacteria contain diazotrophic organisms capable of establishing associations with plant roots [2]. As for other sugarcane soils [1, 2], SPV and SMC had less clones affiliated with Alphaproteobacteria, with much abundance of those OTUs in SPS and SFS.

Rhizobiales (Alphaproteobacteria) related to the denitrification processes (*nirS*,* nirK*, and* nosZ* gene clones) in soil [33] was identified to be highly abundant in all biomes analyzed, but some OTUs were mainly shared by SPS and SFS (Table 3). In forest soils converted to agriculture and fertilized with nitrogen, the abundance of groups belonging to this order decreased. Due to the great importance of this bacterial group regarding soil quality, the combination of rhizobial inoculants and nitrogen-fixing plants is recommended to improve nitrogen fixation and restore ecosystems that have suffered disturbances related to cultivation [34]. In our analysis, the introduction of vinasse in SPV and SMC reduced the number of OTUs compared to SPS and SFS; however, Rhizobiales exhibited considerable frequency in SPV, which ensures the quality of the soil in relation to the nitrogen cycle that is extremely important to the development of cultivation sugarcane in this area. Acidobacteria had increased abundance relative to other bacterial phyla in more acidic soils [14]. Although SPS had a lower pH than other soils, Acidobacteria were detected as the third most common phylum in SPS and second only in SPV (Table 2). This shows that, in addition to the lowering pH, the complex vinasse components (high content of organic matter, glycerol, minerals, etc.) can act as positive selective factors that favor an increased abundance of species of Acidobacteria phylum compared to other environments (Figure 1) [7]. As in other soils with pH of approximately 4.9 [35–37], SPV had a higher abundance of the species related to subdivisions 3 (32 OTUs) and 4 (22 OTUs) of Acidobacteria (Figure 1), probably selected by a pH reduction related to vinasse.

Members of the phylum Actinobacteria are important decomposers of organic matter found in agricultural soils and forests [11] because of the ability of its members to produce cellulase, hemicellulases, chitinases, glucanases, and amylase, which are able to decompose plant cell walls [15]. When compared to other more abundant phyla, Actinobacteria consist of a few species; its members have high phenotypic diversity, including uncultivated aerobic heterotrophs [30].

Each of the ecosystems has different sources for the replacement of organic matter such as natural vegetation debris (litter in SFS), crop residues from other crops (SPV, SPS, and SMC), and vinasse (SPV). Probably because of this wide variation in organic matter inputs, the Actinobacteria phylum was detected as one of the three most abundant phyla in these environments, appearing to be abundant in SPV. We also emphasize that almost all of the OTUs related to Actinobacteria from the four biomes were similar to bacterial strains commonly isolated from soil, showing many well-known characterized strains (Figure 2).

In soil, the population growth of heterotrophic bacteria is attributed to the presence of vinasse [10]. Our results agree with these predictions because many heterotrophic bacterial groups were identified within 11 phyla identified in SPV, such as Proteobacteria, Acidobacteria, Actinobacteria, Firmicutes, and Bacteroidetes (Table 2). These microorganisms have been described as promoters of soil quality control, where the vinasse has potential to select bacterial taxa such as* Actinomycetes* [7]. In SPV, Actinobacteria (or* Actinomycetes*) had a higher frequency of OTUs related to the Micromonosporaceae, Nocardioidaceae, Mycobacteriaceae, and Pseudonocardiaceae families (partially shown in Figure 2), that seem to be more favored in SPV than in the other three biomes.

### 4.2. Richness and Diversity of the Four Biomes

In relation to previous studies in subtropical forest soils [14], our ecosystems were classified as having high diversity. According to analyses of indirectly accessed soil microorganisms in the presence of vinasse [7, 10], SPV had a higher bacterial diversity than any other biome analyzed in this study, showing the potential of vinasse to nurture plenty of bacterial species in the soil (Table 4 and Figure 1). Forest soils have less diversity of bacterial taxa than agricultural soils, a fact related to the increase or absence of the abundance of certain bacterial taxa in forest soils for agricultural soils [11]. For these reasons, SFS showed less diversity in bacterial species than the other three soils studied, with the exception of SMC (Table 4), probably due to the very heterogeneous conditions to which that environment was subjected.

### 4.3. Differences in the Structure of Bacterial Communities

Changes caused by different types of natural and/or anthropogenic disturbances on agricultural land and in forests cause significant changes in the composition of the bacterial communities [11] including the effects of sugarcane transgenic cultivation and herbicide application on bacterial communities in soil [1]. The Libshuff test demonstrated these perturbations, and significance of *P* < 0.01 (data not shown) was obtained between the four environments. Even with differences in the composition of the bacterial communities, SPF and SFS shared some bacterial taxa (Table 3), indicating that although subject to different management practices, some taxa that compose the bacterial communities in these soils showed physiological and metabolic similar characteristics (Figure 3), as OTUs belonging to order Rhizobiales.

Those differences between the bacterial communities for the four environments directly reflected the observed bacterial species. In particular, the species of Proteobacteria were the most diverse and environmental specific, in which the clones showed similarity with bacterial isolates from different important environmental physiological profiles (Table 5). In SPV,* Azohydromonas lata* (SPV212),* Cupriavidus metallidurans* (SPV150), and* Bradyrhizobium valentinum* (SPV115) are generally associated with nitrogen fixation and with great potential for use in bioremediation of sites contaminated with heavy metals [38–40].* Acidiphilium cryptum* (SMC121) identified in SMC is associated with more acidic environments and possesses the ability to reduce Fe^+3^ and Fe^+2^, denoting participation in the iron cycle in soils [41]. SPS presented* Caulobacter vibrioides* (SPS230) and* Rhodoplanes cryptolactis* (SPS052) participating in the carbon sources metabolism arising from organic matter in the soil, besides nitrogen fixation and growth promotion in plants [42, 43]. This comparison shows that, in nonadded vinasse soil (SPS), species with various metabolic profiles and more adapted to plant-interaction seems to be favored. However, the SPV and SMC soils present greater availability of macro- and micronutrients than other soils probably due to the wealth of organic matter and minerals present in vinasse [7–10], with exceptions of nitrogen and iron. For this reason, to address the lack of these nutrients, we speculate that bacteria are playing major important ecological roles in nitrogen and iron cycles and they are even favored in the presence of vinasse.

## 5. Conclusion

The vinasse increases bacterial diversity in soil and promotes favoring species participating in the nitrogen and iron cycle. Species subdivisions 3 and 4 of Acidobacteria were more abundant in soil in which vinasse was applied. Species of families belonging to Actinomycetales were more diverse in soil in which vinasse had been applied for sugarcane cultivation than in other soils.

## Figures and Tables

**Table 1:** Physicochemical properties of the soil from four biomes studied in this work at depth of 0–20 cm.

Soils	pH	OM	P resin	K	Ca	Mg	H + AL	SB	*T*	*V*
	CaCl_2_	g/dm^3^	mg/dm^3^	mmol_c_/dm^3^						%
SPV	5.5	20	33	9.8	44	19	31	72.8	103.8	70
SMC	6.4	19	26	10.3	106	26	12	142.3	154.3	92
SPS	4.8	10	9	1	13	7	28	21	49	43
SFS	5.2	23	31	1.1	39	22	38	62.1	100.1	62

pH: hydrogen potential; OM: organic matter; K: potassium; Ca: calcium; Mg: magnesium; H + AL: exchangeable acidity or potential acid; SB: sum of bases; *T*: Cation Exchange Capacity (CEC) effective at pH 7.0; *V*: percentage of base saturation; mmol_c_/dm^3^: millicentimole per cubic decimeter.

SPV: soil with stillage fertigation and sugarcane cultivation; SMC: heterogeneous soil deposited on the stillage master channel; SPS: soil by sugarcane planting without stillage fertigation; SFS: native forest soil located near areas of sugarcane cultivation.

**Table 2:** Bacterial phylotypes distribution in clone libraries of 16S rRNA gene as affiliation by Classifier algorithm available on RDP II using cutting 80% confidence.

Phylogenetic groups	Clone library (%)^a^				
	SPV	SPS	SMC	SFS	Total
Proteobacteria	30.3	62.9	40.0	59.6	48.2
Alphaproteobacteria	22.1	56.8	19.3	55.0	38.3
Betaproteobacteria	5.0	1.8	0.4	0.4	2.0
Gammaproteobacteria	—	0.7	6.4	—	1.8
Deltaproteobacteria	0.4	—	—	0.4	0.2
Unclassified Proteobacteria	2.9	3.2	13.9	3.6	6.0
Acidobacteria	28.2	8.9	13.2	6.1	14.1
Actinobacteria	6.4	20.7	13.9	14.6	13.9
Firmicutes	2.9	0.7	7.1	—	2.7
Gemmatimonadetes	1.4	0.7	0.3	2.5	1.3
Others^b^	11.0	1.0	—	—	2.9
Unclassified bacteria	20.0	5.0	25.0	17.1	17

^a^Abbreviations of the four biomes are shown in Table 1.

^b^Others: Planctomycetes, Nitrospirae, Bacteroidetes, Chloroflexi, Armatimonadetes, Candidatus phyla Saccharibacteria, and Candidatus phyla division WPS-1.

Normalization factor is 280/total, where total is sum of the number of sequences not chimeras. The normalization factor of each environment was multiplied by each taxon observed at the level of phylum and converted to percentage.

**Table 3:** OTUs of Alphaproteobacteria formed with mothur (3% similarities) and identified by MegaBlast and Classifier from RDP II.

OTU formed	Shared by clones environments	Description based on RDP II classification^*∗*^	Results in MegaBlast		
			Accession (16S rRNA database)	Similarities in MegaBlast (%)	Genus
Otu001	SFS004, SFS058, SFS189, SPS048, and SPS052	Order Rhizobiales and family Hyphomicrobiaceae unclassified (SFS004, SFS058, SFS189, and SPS052) and *Rhodoplanes* (SPS048)	NR_134225.1	97	*Variibacter gotjawalensis*

Otu004	SPS074, SPS064, SPS075, SFS037, and SPV011	Order Rhizobiales (SPS075 and SFS037) and family Hyphomicrobiaceae unclassified (SPS074, SPS064, and SPV011)	NR_125638.1	96	*Bradyrhizobium valentinum*

Otu011	SFS036, SFS027, and SPS171	Order Rhizobiales (genus *Nitrobacter* SFS036 and SFS027) and Bradyrhizobiaceae unclassified (SPS171)	NR_125638.1	100	*Bradyrhizobium valentinum*

Otu012	SFS122, SFS193, and SPS012	Order Rhizobiales and Xanthobacteraceae unclassified (SFS122) and Rhizobiales unclassified (SFS193 and SPS012)	NR_043515.1	95	*Pseudolabrys taiwanensis*

Otu018	SFS057 and SPS023	Family Bradyrhizobiaceae (SPS023) and genus *Bradyrhizobium* (SFS057)	NR_133987.1	99	*Bradyrhizobium neotropicale*

Otu019	SFS069 and SPS024	Order Sphingomonadales (SPS069) and family Sphingomonadaceae (SPS024)	NR_133862.1	97	*Sphingomonas daechungensis*

Otu039	SFS064 and SPS173	Order Rhizobiales (SPS173) and Methylocystaceae unclassified (SFS064)	NR_134156.2	94	*Rhodoplanes oryzae*

Otu052	SFS025 and SPS203	Order Rhizobiales (SPS025) and Hyphomicrobiaceae unclassified (SPS203)	NR_134225.1	96	*Variibacter gotjawalensis*

Otu067	SFS071 and SPS080	Family Sphingomonadaceae (SPS080) and Sphingomonadales (SPS071)	NR_074280.1	96	*Sphingopyxis alaskensis*

Otu068	SFS182 and SPS078	Order Rhizobiales (SPS078) and Rhizobiales unclassified (SFS182)	NR_112190.1	94	*“Rhodoplanes cryptolactis”*

^*∗*^Based on 95% confidence threshold in Classifier.

**Table 4:** Diversity and richness rates observed for each bacterial community present in the soils analyzed. The results were based on 16S rRNA gene of the bacterial clones obtained from 4 environments^a^.

Indices	Environments analyzed^b^			
	SPV	SMC	SPS	SFS
^c^ *S*	188	168	176	179
^d^ *N*	193	193	193	193
^e^Evenness	0.98	0.97	0.97	0.97
^f^Richness	0.93	0.76	0.83	0.85
^g^Shannon	5.23 (5.33)	5.06 (5.17)	5.13 (5.24)	5.14 (5.25)
Simpson	0.0003	0.0019	0.0011	0.0012
Chao 1	2,963 (1,488–6,109)^h^	1,039 (645–1,758)	1,262 (765–2,178)	2,231 (1,172–4,415)
Singletons	179 (193)^i^	145 (191)	161 (193)	163 (191)

^a^Calculations were based on OTUs formation based on evolutionary distance of ≤ 0.03. ^b^Abbreviations are shown in Table 1. ^c^ *S* is number of OTUs observed. ^d^ *N* is sequences number. ^e^Evenness is *H*/*H*_max_ [44]. ^f^Richness is (number of OTUs singletons − 1)/log⁡*N*. The observed value/maximum possible is informed. ^g^Maximum value observed for Shannon (*H*). ^h^Confidence intervals (95%) for Chao 1. ^i^Maximum value observed for singletons.

**Table 5:** OTUs related to bacterial orders in four soil biomes (cutoff of 95% confidence in RDP II Classifier) and identity by MegaBlast.

Sequence	Genus	Accession GenBank	Similarities (%)	Description	References
SPV212	*Azohydromonas lata*	NR_041244.1	94	Nitrogen fixation in different soils	[38, 45]
SPV150	*Cupriavidus metallidurans*	NR_027607.1	97	Chemolithotrophic bacteria common in agricultural soils and showing potential for bioremediation of soils contaminated with Cd, Zn, and Cu, among others	[39, 46]
SPV115	*Bradyrhizobium valentinum*	NR_125638.1	97	Nitrogen fixation in different soils	[40]
SPS230	*Caulobacter vibrioides*	NR_037099.1	97	Utilizes various sources of carbon and has potential for application in selenium solubilizing	[42, 47]
SPS052	*“Rhodoplanes cryptolactis”*	NR_112190.1	96	Nitrogen fixation, organic matter decomposition, and growth promotion in plants	[43]
SMC121	*Acidiphilium cryptum*	NR_119294.1	91	Iron reduction (Fe^+3^ to Fe^+2^) in acidic conditions	[41]
SFS043	*Sphingosinicella microcystinivorans*	NR_040927.1	96	Relatively new genus which has the capacity to degrade polyaromatic hydrocarbons	[48]
